# Hybrid Methods in Iron-Sulfur Cluster Biogenesis

**DOI:** 10.3389/fmolb.2017.00012

**Published:** 2017-03-13

**Authors:** Filippo Prischi, Annalisa Pastore

**Affiliations:** ^1^School of Biological Sciences, University of EssexColchester, UK; ^2^Maurice Wohl Institute, King's College LondonLondon, UK; ^3^Molecular Medicine Department, University of PaviaPavia, Italy

**Keywords:** frataxin, NMR, molecular complexes, small angle X-ray scattering, structural biology, iron-sulfur cluster machinery, hybrid methods

## Abstract

Hybrid methods, which combine and integrate several biochemical and biophysical techniques, have rapidly caught up in the last twenty years to provide a way to obtain a fuller description of proteins and molecular complexes with sizes and complexity otherwise not easily affordable. Here, we review the use of a robust hybrid methodology based on a mixture of NMR, SAXS, site directed mutagenesis and molecular docking which we have developed to determine the structure of weakly interacting molecular complexes. We applied this technique to gain insights into the structure of complexes formed amongst proteins involved in the molecular machine, which produces the essential iron-sulfur cluster prosthetic groups. Our results were validated both by X-ray structures and by other groups who adopted the same approach. We discuss the advantages and the limitations of our methodology and propose new avenues, which could improve it.

## Introduction

Biophysical approaches that make the combined and integrated use of different methodologies are named “hybrid techniques.” Their use in Structural Biology has rapidly caught up in the last ca. 20 years (Sunnerhagen et al., 1996; Improta et al., 1998; Putnam et al., 2007; Tuukkanen and Svergun, 2014; Delaforge et al., 2015; Kachala et al., 2015; Milles et al., 2015; Sali et al., 2015; Prischi and Pastore, 2016; Venditti et al., 2016). A particularly useful application of hybrid techniques is the use of a combination of high and low resolution techniques which first target the local structure of a molecule (a domain or a complex component) and then reconstruct the full picture of the assembly (the so-called cut-and-paste approach) (Grishaev et al., 2005, 2008; Parsons et al., 2008; Deshmukh et al., 2013). Hybrid methods have, for instance, been successfully introduced to gain structural insights of complexes with different sizes which would be unaffordable if approached by only one technique (Wüthrich, 2001). One of the very first examples of hybrid methods was our study based on a combination of small angle scattering (SAXS) and nuclear magnetic resonance (NMR) to approach the arrangement of the domains of titin, a giant muscle modular protein containing more than 300 copies of two all-β sequence motifs, the fibronectin type 3 and the immunoglobulin-like modules (Improta et al., 1998). More recently, Michael Sattler and co-workers extended the approach to the study of RNA-protein interactions (Gabel et al., 2006; Madl et al., 2011; Huang et al., 2014). In 2010, we implemented a robust methodology which brings together NMR, SAXS, *in site* directed mutagenesis, ITC and other techniques to study weak complexes. This methodology has proven particularly effective for proteins of the iron-sulfur (FeS) clusters biogenesis machine, a highly conserved and essential metabolic pathway (Zheng et al., 1998). These proteins share important features which make particularly useful the application of hybrid methods to their study: all the components of this cellular machine (i) tend to form transient interactions, making co-purification of the complexes difficult to impossible; (ii) have different binding affinities from each other; (iii) compete for the same binding sites; (iv) have different likelihood to crystallize, which often results in proteins forming crystals alone and not as part of the complex. In addition, many of the complexes are, although relatively large for NMR studies, too small for cryo-electron microscopy studies (Nogales and Scheres, 2015). As a result, high-resolution structures of most of these protein complexes are still not available. Here, we review our approach, discuss its successes and clarify the limitations. We also suggest ways to circumvent specific problems.

## The paradigmatic example of the iron-sulfur cluster biogenesis components

**Figure 1:**
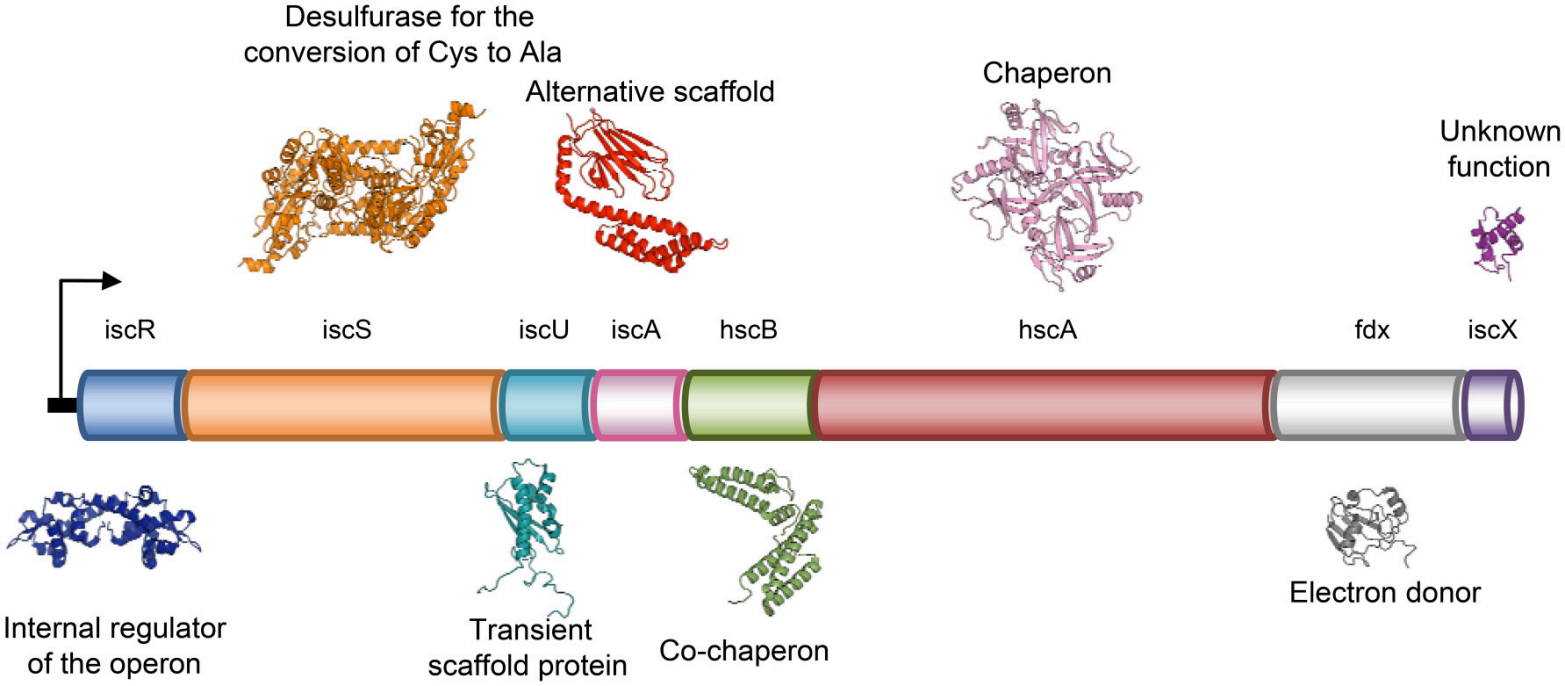
**Schematic representation of the genetic organization of the bacterial ISC system in operon**.

Present ubiquitously in nearly all life forms, FeS clusters are protein inorganic prosthetic groups involved in a multitude of biological functions, such as electron transfer, gene expression regulation, thiolation, photosynthesis, nitrogen fixation, metal trafficking, substrate binding, DNA repair/replication and RNA modification (Johnson et al., 2005; Mettert and Kiley, 2015). FeS clusters are formed from iron ions and inorganic sulfide. Due to the toxic nature of these elements, formation of intracellular FeS clusters does not occur spontaneously, but all organisms have evolved protein machineries for the production of clusters. The FeS cluster assembly (ISC) system is a highly conserved factory found both in prokaryotes and eukaryotes and capable of providing FeS clusters to a wide range of apo-proteins. In particular, the eukaryote ISC machine is found in the matrix space of mitochondria and is distinct from the system that produces the clusters in the cytosol (Lill, 2009; Rouault, 2015). In *E. coli*, which is most studied as a model system because of its lower complexity, the ISC machine is composed of eight genes clustered in an operon, *iscRSUA-hscBA-fdx-iscX* (Takahashi and Nakamura, 1999; Figure 1). The operon is controlled by IscR, a transcriptional repressor (Schwartz et al., 2001) followed, in the order, by genes coding for a cysteine desulfurase (IscS) (Schwartz et al., 2000), a scaffold protein upon which clusters are built (IscU) (Agar et al., 2000), an A-type carrier with unclear function (IscA) (Krebs et al., 2001; Ollagnier-de-Choudens et al., 2001), a co-chaperone/chaperone system that is thought to facilitate cluster transfer from IscU to the final acceptor (hscA and hscB) (Chandramouli and Johnson, 2006), an electron donor ferredoxin (Fdx) (Yan et al., 2013b) and a protein with unknown function (IscX or YfhJ) (Pastore et al., 2006). This system constitutes the so called core assembly machine. The formation of FeS clusters by the core machine starts with the production of S^0^ from L-cysteine by IscS, followed by reduction of S^0^ to S^2−^ by Fdx (Yan et al., 2013b) and ends with the incorporation of Fe^2+^ or Fe^3+^ and formation of a [2Fe-2S] cluster on IscU (Agar et al., 2000). It is still unclear how the iron is delivered to the system.

Among these proteins, the crucial ones are IscS (NFS1 in human) and IscU (ISCU in human). IscS is a pyridoxal 5′-phosphate (PLP)-dependent desulfurase. PLP is not only necessary for the catalysis of L-Cys to L-Ala, but is also important for structural stabilization. CD spectra of recombinant IscS without PLP revealed in fact that the protein is completely unfolded, albeit proteolytically stable and not prone to aggregation (Prischi et al., 2010b). IscU, a 10 kDa protein, is predominantly in a monomeric state in solution and binds to IscS to accept the sulfur, which will form the clusters. The function of IscS and IscU are regulated by the protein frataxin (FNX). This is an essential protein highly conserved both in prokaryotes (where takes the name of CyaY) and eukaryotes where it is present in mitochondria. FXN was first identified for its connection to Friedreich's ataxia (Campuzano et al., 1996), a progressive neurodegenerative disease caused by an expansion of a GAA trinucleotide repeat within the first intron of the FXN gene, which results in reduced levels of FXN (Campuzano et al., 1996, 1997). Studies on the yeast frataxin homolog (YFH1) helped to understand that reduced levels of FXN causes loss of function of FeS cluster containing enzymes, increased amount of free radicals and iron deposits in mitochondria (Babcock et al., 1997; Foury and Cazzalini, 1997; Koutnikova et al., 1997; Rötig et al., 1997). Proteins from the FXN family bind weakly ferrous ions (Kd 4 μM) and ferric ions (Bou-Abdallah et al., 2004). These features are strongly conserved: human FXN is able to bind Fe^2+^ and Fe^3+^ in a similar way (Yoon and Cowan, 2003). Ability to weakly bind iron could be in agreement with the hypothesis that the protein functions as an iron chaperone, but the way FXR binds iron is unusual. The FXN fold, which is composed of two α-helices packed against an anti-parallel β-sheet (Cho et al., 2000), does not share any similarity with any other known iron binding proteins, like ferritins, ferredoxins or hemoglobins (Harrison and Arosio, 1996). It is also unusual that iron coordination occurs solely through carboxylate residues and no conserved histidine, cysteine, or tyrosine - residues usually found in iron binding motifs - are present in frataxins (Nair et al., 2004). Finally, cation binding is highly unspecific since, in addition to iron, frataxins bind to diamagnetic Ca^2+^, Zn^2+^, Lu^3+^, and paramagnetic ions Mn^2+^, Co^2+^, Gd^3+^, Eu^3+^, and Yb^3+^ (Nair et al., 2004). Twenty years have passed since these initial studies which have made clear that FXN is connected to FeS cluster formation, but the exact function of FXN remains elusive. Different theories have been proposed: (i) FXN is the iron chaperone that delivers Fe^2+^ or Fe^3+^ to IscU (Yoon and Cowan, 2003); (ii) FXN acts as a scavenger that is able to sequester mitochondrial iron through formation of high-molecular-weight aggregates and to maintain it in a bioavailable form (Adamec et al., 2000). We have proposed a third hypothesis which is currently the most accredited: (iii) FXN acts as an iron sensor that regulates the amount of FeS cluster formed to match the concentration of the available acceptors (Adinolfi et al., 2009). Our model, which proposes a completely new function of FXN, is based on studies that rely on the demonstration that FXN binds to the IscS/IscU complex in an iron dependent manner (Prischi et al., 2010a). To gain information on this ternary complex, we adopted a hybrid approach, which relied on NMR, SAXS, site directed mutagenesis, molecular docking and molecular dynamics simulations.

## The development of a hybrid method

**Figure 2:**
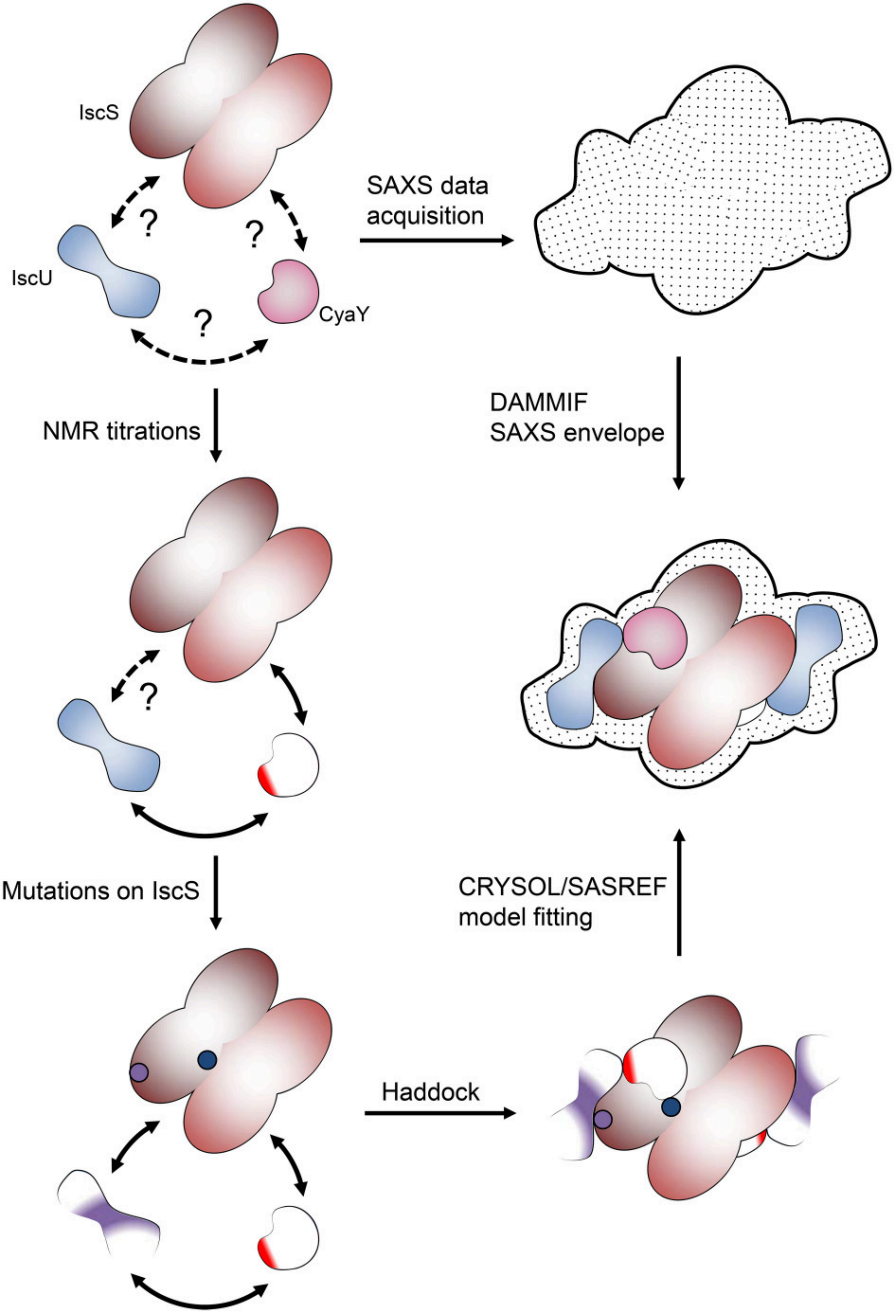
**Pipeline of the hybrid methodology described in this review**. At the beginning we only know that proteins interact but do not know anything about their mutual orientation. NMR allowed us to map the surface of interaction of the lower molecular weight proteins. The surface involved in interaction on IscS was identified by a combination of mutations and SAXS experiments.

The rationale of our hybrid method develops through the following logic steps (Figure 2):

### Step 1: identifying the ISC interactome

The network of interactions between IscS, IscU, and CyaY was probed by NMR spectroscopy. We exploited the well-known concept that the spectrum of a molecule is very sensitive to the chemical environment. Protein-protein interaction cause changes in the chemical environment of the reporter nucleus. This means that titration of a protein with another molecule results in shifts in the position of some or all resonances in the spectrum or Chemical Shift Perturbation (CSP) (Roberts, 1993; Zuiderweg, 2002), which can then be used to map the regions involved in the interaction. Typically, we titrated a ^15^N labeled component of the *isc* operon with another un-labeled protein. However, the resonance line widths depend inversely on the tumbling time (Bloembergen et al., 1948) and, thus, the larger the complex, the broader are the line widths up to spectral disappearance. Many of the ISC components have sizes well within the limits of NMR observation except for IscS, which is an obligate dimer of 90 kDa, and the chaperone HscA. This meant that we could alternatively titrate the low size proteins (i.e., adding unlabeled protein A into ^15^N labeled protein B and, viceversa, unlabeled protein B into ^15^N labeled protein A) and map the interacting site on both proteins. The case was quite different when adding the 90 kDa IscS to a smaller component. In this case the result would strongly depend on the regime of exchange of the complex.

**Figure 3:**
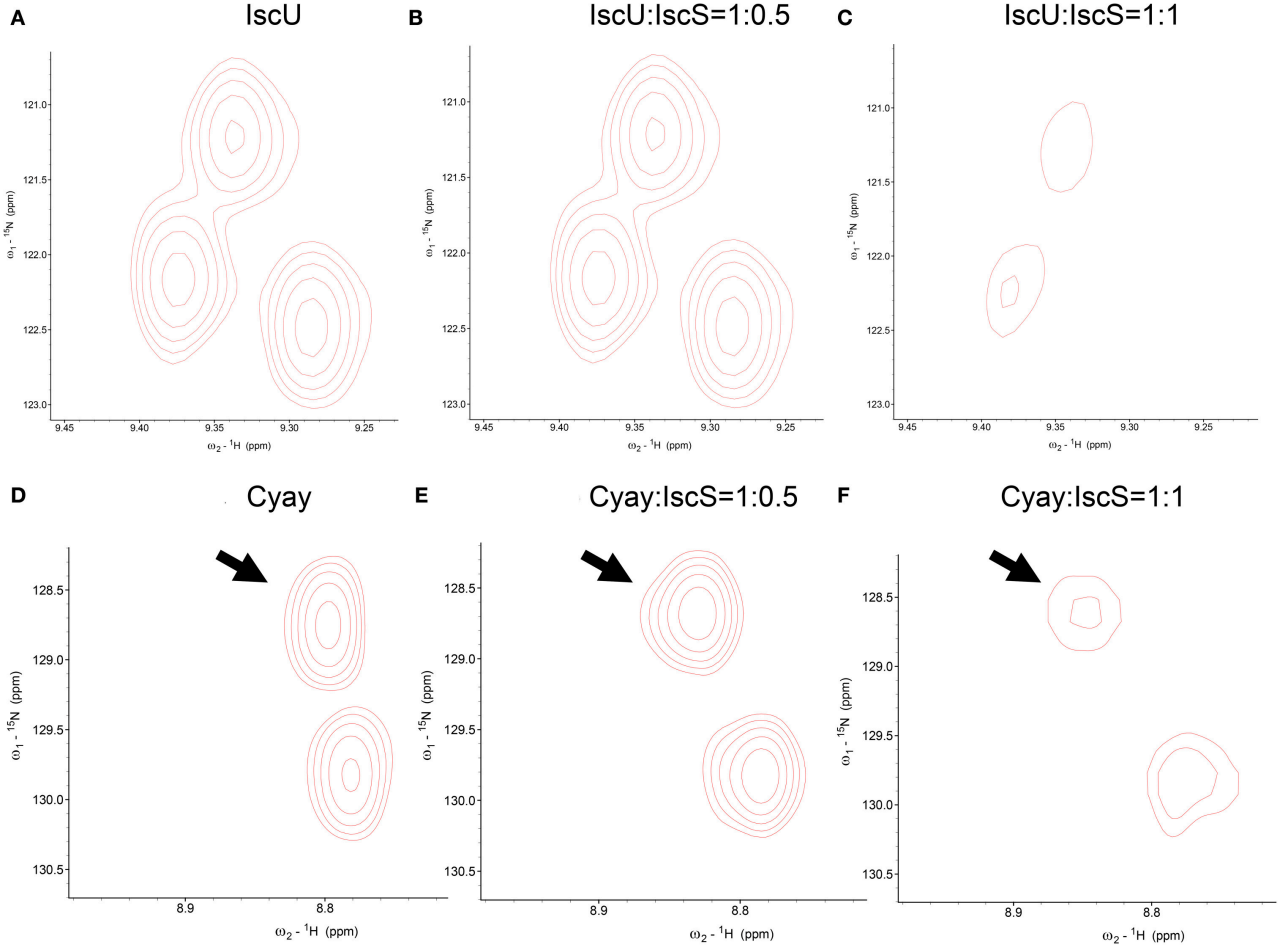
**Comparison among close-ups from HSQC spectra of 15N uniformly labeled IscU. (A)** From left to right, IscU alone; **(B)** IscU in the presence of unlabeled IscS at a molar ratio of 1:0.5; **(C)** The same as in **(B)** but at a molar ration of 1:1. No detectable CSP of IscU peaks and concomitant disappearance of the IscU spectra when saturated with IscS suggest that the process is under an intermediate-to-slow exchange regime in the NMR time range. For comparison, The spectra were all recorded at 25°C and 600 MHz and visualized using the same signal to noise ratio. **(D)** CyaY alone; **(E)** CyaY in the presence of unlabeled IscS at a molar ration of 1:0.5; **(F)** The same as in **(E)** but at a 1:1 molar ratio. The spectra were all recorded at 25°C and 600 MHz and visualized using the same signal to noise ratio.

The most common NMR experiment used to measure CSP is the two-dimensional ^15^N heteronuclear single-quantum coherence NMR ([^1^H,^15^N]-HSQC NMR), a method that allows the detection of correlations between ^15^N nucleus and ^1^H nucleus which are covalently bound. Titration of IscS into ^15^N labeled IscU caused complete disappearance of IscU signal from the [^1^H,^15^N]-HSQC NMR spectra at a 1:0.7 IscU:IscS molar ratio (Prischi et al., 2010b) without previous CSP. Absence of detectable CSP for these titrations and disappearance of the IscU signal indicates binding but also suggests that the process is under an intermediate-to-slow exchange regime in the NMR time range (Figures 3A–C). The exchange regime is the rate *k*_*ex*_ at which a nucleus switches from one conformation to another (in this case a “free state” to a “bound state”). The NMR linewidths depend on the populations of each state, the relative values of the exchange rate *k*_*ex*_ and the chemical shift difference Δν. In the slow exchange regime (*k*_*ex*_ < < |Δν|), signals from both states are observed at their distinct chemical shifts, intensities and linewidths; if the regime is fast (*k*_*ex*_ >> |Δν|), a single peaks will be observed at the chemical shift between free and bound conformations weighed according to the populations; if it is in an intermediate regime (*k*_*ex*_ ≈ |Δν|), a single peak is observed between the two states but due to the presence at the same time of the free state and the bound state, the resulting resonance is broadened (Kleckner and Foster, 2011). In our case IscU alone corresponds to the “free state”, while the IscU-IscS complex is the “bound state”. Since the spectra are completely unperturbed until we reach a 1:1 ratio IscU:IscS, we can deduce that this is not in a fast exchange regime in the NMR time range and we can deduce that the process is an intermediate-to-slow exchange regime. We would expect then to see peaks for both the free state and the bound one. However, the high molecular weight of the complex causes that the bound state is outside the limit of NMR observation and we do not observe it. This did not allow us to map the interaction surface of IscU on IscS, a problem often observed in the NMR studies of complexes. We also did not observe CSP when titrating directly IscU and CyaY in the absence of IscS but in this case the spectra of the two proteins, individually labeled in turn, where completely unaffected. This meant no direct interaction in contrast with studies carried out on the human and yeast proteins, where a direct interaction between the scaffold protein and FXN was observed (Yoon and Cowan, 2003; Correia et al., 2009; Leidgens et al., 2010), suggesting a different behavior of the bacterial proteins. The difference could perhaps be ascribed to the lack of the N-terminal extension which, in eukaryotes, is part of the mitochondrial signal and absent in prokaryotes. Finally, when titrated with IscS, the spectrum of CyaY remains visible and shows clear CSP, which allowed us to map the interaction on a specific surface (Figures 3D–F). CyaY interacts with IscS using a negatively charged surface area localized on α1, β1 and α1β1 and β1β2 loops (Adinolfi et al., 2009). Interestingly, this negatively charged surface is the same involved in iron binding (Yoon and Cowan, 2003; Bou-Abdallah et al., 2004; Nair et al., 2004). We then tested for competition between CyaY and IscU binding on IscS by [^1^H,^15^N]-HSQC NMR spectra titrating ^15^N labeled IscU with up to an equimolar amount of unlabeled IscS with unlabeled CyaY. Presence of competition should cause dissociation of ^15^N labeled IscU from IscS, resulting in the reappearance or increase of the NMR signal, in a way proportional to the amount of competitor added. We did not observe competition with IscU (Adinolfi et al., 2009; Prischi et al., 2010a). We could thus conclude that both CyaY and IscU bind to IscS, but not each other and obtain the surface of interaction on CyaY from NMR only.

**Figure 4:**
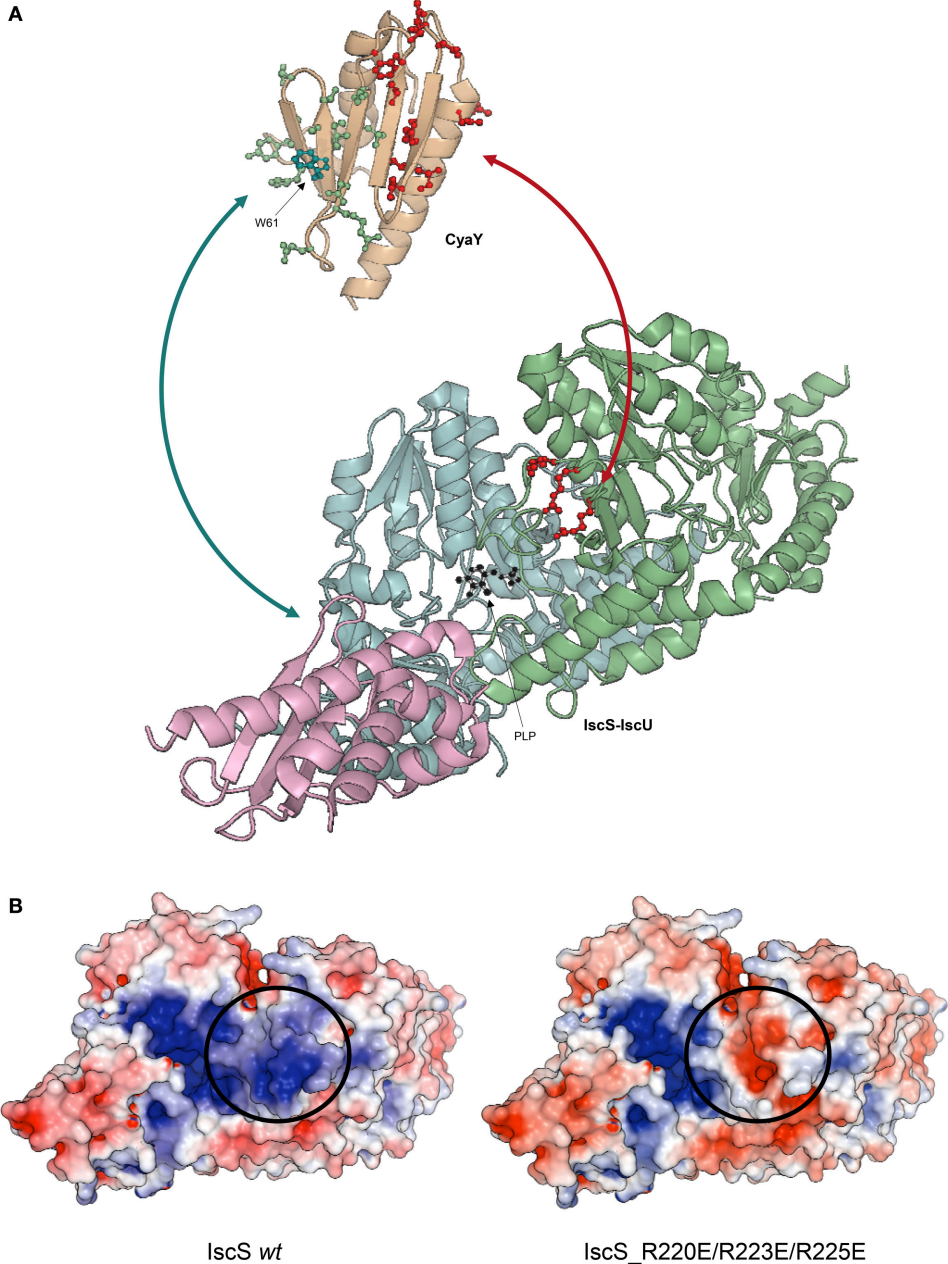
**Ribbon representation of CyaY and IscS-IscU interactions. (A)** IscU is shown in pink, while IscS monomers are colored in pale cyan and light green with side chains of residues mutated indicated explicitly: R220E/R223E/R225E (red). PLP in IscS active site is shown in black. Side chains of CyaY residues exhibiting CSP are explicitly shown: residues interacting with IscS are in red (Trp14, Leu15, Glu19, Asp22, Asp23, Trp24, Asp25, Asp27, Ser28, Asp29, Ile30, Asp31, Cys32, Glu33, Ile34, Leu39, Thr42, Phe43, Glu44, and Gly46), while residues interacting with IscU are in green (Thr40, Ile41, Lys48, Ile50, Asp52, Arg53, Glu55, Trp61, Leu62, Ala63, Thr64, Gln66, Gly68, Tyr69, and His70). **(B)** Electrostatic surface of unbound Iscs. The circle indicates the position of the positively charged residues involved in binding.

CSP data did rule out the presence of a direct interaction or competition between CyaY and IscU, but this did not automatically exclude binding between the two when in the presence of IscS. We titrated ^2^H, ^15^N double-labeled CyaY with unlabeled IscU and IscS up to a 1:1:1 molar ratio. ^2^H labeling reduces spin-spin relaxation, a parameter inversely proportional to the linewidths of the resonance in the spectrum. This results in narrower linewidths and thus provide higher resolution (Gardner and Kay, 1998). We observed a new set of spectral perturbations, which we attributed to a direct contact of the residues involved with IscU. Once mapped onto CyaY structure, these residues clustered on the anti-parallel β-sheet surface of CyaY (Figure 4A). In particular, the conserved Trp61 in CyaY was found to be involved in the interaction with IscU. These data are in agreement with studies on human FXN, where it was shown that the exposed side chain of Trp155 (equivalent to CyaY Trp61) is indispensable for FXN-ISU binding (Correia et al., 2009; Leidgens et al., 2010).

To map the surface of interaction on IscS we used site directed mutagenesis. We designed mutations of IscS targeting solvent exposed residues. We aimed to abolish interaction with ISC components, while keeping IscS stable and functional. We titrated ^15^N labeled IscU and CyaY with five different IscS mutants, i.e., IscS_R220E/R223E/R225E, IscS_I314E/M315E, IscS_K101E/K105E, IscS_E334S/R340S and IscS_R39E/W45E (Prischi et al., 2010a). IscS_R220E/R223E/R225E triple-mutant, in which a positively charged patch formed mainly by arginines close to the dimer interface was inverted in charge, does not bind CyaY (Figure 4B; Prischi et al., 2010a). This strongly supported the assumption that binding between these proteins is driven by electrostatic interactions and is in agreement with our competition studies. Differently, in IscS_I314E_M315E we inserted two charged residues into an uncharged/hydrophobic patch. This mutant has a reduced affinity for IscU, which, due to a change in the exchange regime, caused chemical shift perturbation of the ^15^N-labeled IscU HSQC spectrum. This not only allowed us to identify IscS interacting surface, but also to identify the IscU residues involved in IscS binding (Figure 4A; Prischi et al., 2010a). All other IscS mutants behaved like the wild type in titration experiments and provided us with solid controls (Prischi et al., 2010a).

### Step 2: restrained docking simulation

To gain a visual impression and understand the relative orientation of proteins in the complex, we generated models of the central ISC machine using NMR restrained molecular docking simulations. We used the docking software HADDOCK (Dominguez et al., 2003). HADDOCK can incorporate NMR or other distance restraints and implement them as “ambiguous interaction restraints” (AIRs). The software forces the protein interfaces to come together without imposing a particular orientation. Using the AIRs that we determined experimentally during Step 1, we obtained different families of complexes, which differed by details but all reported IscU bound on the opposite tips of the IscS dimer, roughly close to the N-terminus. CyaY was instead consistently located near the cavity that contains the active site of IscS, spatially close but not overlapping with IscU. While these results could have been sufficient for having a first rough model of the IscS-IscU-CyaY complex, we felt that further validation was needed to confirm independently the relative positions of the three proteins.

### Step 3: validation through SAXS data

**Figure 5:**
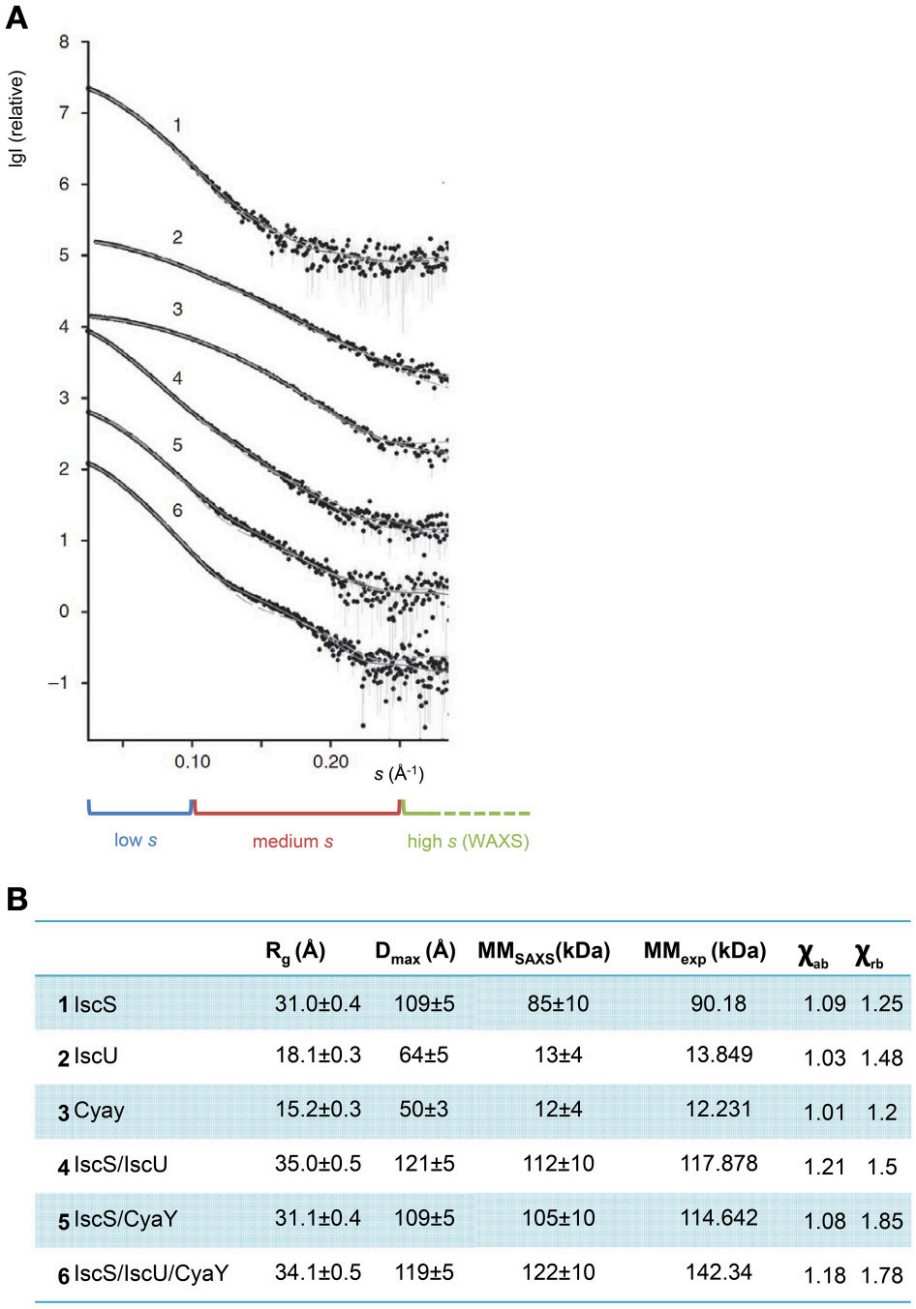
**SAXS profiles of the complexes. (A)** The X-ray scattering patterns from IscS (1), IscU (2), CyaY (3) binary complexes IscS/IscU (4) and IscS/CyaY (5) and ternary complex IscS/CyaY/IscU (6). Plots display the logarithm of the scattering intensity as a function of momentum transfer (*s*). At the bottom it is highlighted with a blue box the low *s* region of the SAXS curve, in red the medium and in green the high. The experimental data are displayed as dots with error bars, the scattering from typical ab initio models computed by DAMMMMIF as full lines and the calculated curves from the high-resolution (for proteins alone) and rigid body models (for complexes) computed by CRYSOSOL/SASREF as dashed lines. The successive curves are displayed down by one logarithmic unit for clarity (figure adapted from Prischi et al., 2010a). **(B)** Table summarizing SAXS data. R_g_ is the radius of gyration; D_max_ is the maximum size of the particle; MM_SAXS_ is the molecular mass calculated from SAXS data; MM_exp_ experimental molecular mass of the solute and χ_ab_ and χ_rb_ values for the fit curves from *ab initio* models and from high resolution models (for proteins alone) and rigid body modeling (for complexes) using CRYSOL/SASREF, respectively.

The generated models were then experimentally verified and re-scored using SAXS data. SAXS is a solution technique that allows to study the shape, conformation and assembly state of proteins and, more in general, macromolecular complexes (Mertens and Svergun, 2010). Despite being a low-resolution technique, SAXS is well suited for the study of flexible systems and intrinsically disordered proteins (Wang et al., 2011), which are major limitation in X-ray crystallography and cryo-EM. It also allows the study of proteins in solution in nearly-native conditions. SAXS experiments are not time consuming. Recent hardware improvements allow high-throughput studies (Round et al., 2008). A monochromatic X-ray beam is scattered by the protein sample in solution. At low (below 0.1 Å^−1^) and medium momentum transfer (*s*) (between 0.1 Å^−1^ and 0.25/0.3 Å^−1^) scattering angle, we obtain different information about the system (Figure 5A). Above 0.3 Å^−1^ the noise masks the signal and above 0.5 Å^−1^ data are collected at wide angle. This technique is not called SAXS anymore, but WAXS (Wide Angle X-ray Scattering) (Graewert and Svergun, 2013). At low *s* it is possible to extrapolate the radius of gyration *R*_g_, which provide information about the size of the protein (Grant et al., 2015; Kikhney and Svergun, 2015). In order to obtain a reliable *R*_g_, it is important to have a precise measurement of the sample concentration before SAXS measurements. Medium *s* provides information about the shape of the protein. More precisely it is possible to obtain the D_max_, which provides measurement of the maximum dimension of the protein (Svergun, 1992).

From these measurements it is possible to build a low-resolution envelop, which represents the shape of the protein studied. The shape is reliable only when the system under study is monodisperse. In fact, it is always possible to obtain envelops from SAXS data, but poly-disperse samples do not generate envelops that represent the real shape of the protein. For example, in the study of PERK N-terminal domain, the protein was in a dynamic equilibrium between a dimer and a tetramer (Carrara et al., 2015). In this case it is not possible to obtain protein shape information, but SAXS is still informative. It had to be assumed that the resulting shape was a weighted average of dimer and tetramer shapes. The factor of weight had to be obtained from independent techniques, such as analytical ultracentrifuge (AUC), from which the relative populations of PERK dimer and tetramer in solution were estimated. The SAXS curve was then back-calculated using PERK dimer and tetramer crystal structures, weighted according to their relative abundance in solution, and fitted on experimental data. Agreement between experimental and back-calculated SAXS curves provided a confirmation that the PERK oligomeric structures were not a crystallographic artifact, but representative of the oligomeric state of PERK in solution (Carrara et al., 2015).

Luckily, IscS, IscU, and CyaY are all mono-disperse in solution. It was thus possible to obtain reliable information about their shapes from SAXS only. We collected SAXS data for each of the individual components, as well as for the binary (IscS-IscU, IscS-CyaY) and tertiary (IscS-IscU-CyaY) complexes (Prischi et al., 2010a). As previously mentioned, due to the dynamic nature of the Fe-S cluster machinery, the binding affinities of IscU and CyaY for IscS are relatively low: K_dIscU-*IscS*_ = 1.3 ± 0.2 μM and K_dCyaY-*IscS*_ = 18.5 ± 2.4 μM (Prischi et al., 2010a). We were able to isolate IscU-IscS complex using Size-Exclusion Chromatography (SEC) (Prischi et al., 2010b), but not CyaY-IscS and IscS-IscU-CyaY. In these cases we directly mixed proteins in solution prior data collection. Knowledge of relative K_d_ allowed us to estimate the optimal proteins ratios in order to maximize formation of the (Prischi et al., 2010a). It is worth mentioning that, despite not being available when we collected our data, a new methodology, particularly useful when collecting SAXS data on protein complexes, is Size-Exclusion Chromatography in line with SAXS (SEC–SAXS) (Mathew et al., 2004). SEC-SAXS is useful for separating pure systems that are under monomer-oligomer equilibrium or to further purify the sample before SAXS data are collected (particularly indicated for low stability protein which tend to form soluble aggregates). SEC–SAXS is available as a continuous-flow sample delivery option at BioCAT (Advanced Photon Source, U.S.A.) (Mathew et al., 2004), SWING (Soleil, France) (David and Perez, 2009), the SAXS beam line at the Australian Synchrotron, BM29 (ESRF, France), BL23A1 (NSRRC, Taiwan), B21 (Diamond, U.K.) and P12 (DESY, Hamburg) (Blanchet et al., 2015). SEC-SAXS has however limitations and it shouldn't be used as a purification step (Jeffries et al., 2016).

*Ab initio* envelops were generated using the software DAMMIF (Franke and Svergun, 2009). The high-resolution PDB structures 1P3W (Cupp-Vickery et al., 2003) for IscS and 1SOY (Nair et al., 2004) for CyaY were fitted into the SAXS envelops by rigid body modeling. Two different structures were used for IscU: one solved by NMR (PDB ID 1Q48) (Ramelot et al., 2004) and one by X-ray crystallography (PDB ID 2Z7E) (Shimomura et al., 2008). The two structures have a similar overall secondary structure content, but while in the NMR structure the first 25 residues are in a random coil conformation, the crystal structure is more compact and the N-Terminus forms a α-helix which makes contacts with α3 and the α5α6 loop. The quality of fitting of a 3D structure on a SAXS envelop can be visually ascertained, and can be more accurately estimated using the χ^2^ (Svergun, 1999). χ^2^ tells us how well the back-calculated scattering intensity from a 3D structure fits the experimental SAXS data. Fitting of the isolated CyaY and IscS resulted excellent, with a χ^2^ of respectively 1.01 and 1.09 and an estimated molar mass of respectively 12 kDa ± 4 kDa (expected 12.231 kDa) and 85 kDa ± 10 kDa (expected 90.180 kDa) (Figures 5A,B). Of the two structures, 1Q48 fitted better the SAXS data collected for the isolated IscU in agreement with the dynamic nature of isolated IscU in solution (Kim et al., 2009; Prischi et al., 2010b), with a χ^2^ of 1.03 and an estimated molar mass of 13 kDa ± 4 kDa (expected 13.849 kDa) (Figures 5A,B). It is also strongly recommended to check the residuals of the difference between experimental and back calculated SAXS data (i.e., whether these are random and not systematic).

### Step 4: experimental validation of the models

The software DAMMIF (Franke and Svergun, 2009) was used to generate envelops from SAXS data. DAMMIF, a fast version of DAMMIN (Svergun, 1999), carries out an *ab initio* shape determination by simulated annealing using a single phase Dummy Atom Model (DAM). The DAM is represented by a tightly packed group of beads, which mimic, but do not resemble, real atoms. Each bead has a known scattering pattern and the software puts beads together so that the accumulated scattering resembles the experimental data. The software used to generates back-calculated curves and fit them on experimental data is the CRYSOL software (Svergun et al., 1995). CRYSOL requires a 3D structure/object as an input and then, taking into account the contribution for each atom, it evaluates the scattering intensity.

Despite having a 10–20 Å resolution (2π/*s*_*max*_), the SAXS envelops of the binary and tertiary complexes resulted evidently different from those of the single components. We first tested whether SAXS data were sufficient to generate meaningful binary and tertiary complexes using the SASREF software (Petoukhov and Svergun, 2005). SASREF tries to build the quaternary structure of a complex using the structures of the subunits and the solution scattering data. It is particularly useful because it can work with multiple data set(s), which allows working with SAXS data from sub-complexes and creating contrast series. SASREF build the complex structure without steric clashes using a simulated annealing protocol, which minimize differences between the experimental scattering data and the back-calculated SAXS curve of the model being built.

We inputted in SASREF the SAXS data and the high-resolution structures of the single components but the complexes obtained with this approach did not generate reliable models, since they were not in agreement with our binding studies. Instead, we docked our HADDOCK structures into SAXS envelops. HADDOCK models were used to generate back-calculated SAXS curves, which were fitted on experimental data. Based on χ^2^, we selected the best fitting model, which was an experimentally verified model of FeS machinery complexes. Selecting the “best fitting model” could be problematic if two similar HADDOCK models have small orientation differences, which are clearly not distinguishable at SAXS resolution. In this context, HADDOCK is particularly well suited, because it first generates a maximum of 1,000 structures and then groups them according to their relative RMSD. By aligning these generated models using the interface residues of the first molecule, the RMSD (more correctly called interface-ligand RMSD) is calculated for the interface residues (less than 10 Å distance from the first molecule) of the second molecule (Dominguez et al., 2003). In our case, all structures HADDOCK grouped within the same group had RMSD < 7.5 Å. For each group, we used the structures with overall lower energy (evaluated by HADDOCK). Analysis of the binary complexes confirmed that CyaY sits near the IscS dimer interface and the active site, while IscU is located on the periphery of the IscS dimer and is aligned with the long axis of IscS. Accordingly, the IscS-IscU (*R*_g_ = 35 Å, D_max_ = 121 Å) envelop is more elongated than the IscS alone (*R*_g_ = 31 Å, D_max_ = 109 Å), while the IscS-CyaY envelop is more globular (Prischi et al., 2010a; Yan et al., 2013b; Figure 4B).

### Step 5: comparison with X-ray crystal protein complexes

A limitation of this procedure is the absence of a tool to predict/model major structural changes upon formation of a complex. HADDOCK can simulate small conformational changes during the molecular dynamics refinement, but the final model strongly depends on the initial 3D structures provided: HADDOCK assumes a key-in-the-lock model. If a protein has significantly different structures in the free and bound states, HADDOCK (like any other protein docking software) will fail to generate a reliable model. For the IscS-IscU complex, we found that the model generated from HADDOCK did not fit the SAXS envelop as well as the single components did. However, a crystal structure of the IscS-IscU complex (PDB ID 3LVL) (Shi et al., 2010) became available while we were carrying out our studies. This structure is in perfect agreement with our NMR and mutant binding data and fits the SAXS envelop better than the HADDOCK model. This is due to IscU going through a structural rearrangement upon binding, with a formation of a α-helix in the N-terminus, similar to the one seen in 2Z7E (Shimomura et al., 2008). IscU has an optimal orientation for FeS cluster formation, with the surface containing three conserved cysteines pointing toward Cys328 in IscS loop (Shi et al., 2010). The distance between IscS active site and IscU is around 12 Å, suggesting the presence of major conformational changes happening during FeS cluster formation.

We then used 3LVL (Shi et al., 2010) for modeling the tertiary complex, IscS-IscU-CyaY. Interestingly, the model confirmed that, despite not being able to interact between each other directly, CyaY and IscU can interact once bound to IscS. This structure helped us to explain an inhibitory effect of CyaY on FeS cluster formation: enzymatic studies had showed that the tertiary complex is “less dynamic” than the binary ones with CyaY creating an additional anchoring point between IscS and IscU (Prischi et al., 2010a). This is in agreement with the observation that CyaY binding increases the affinity of IscU for IscS thus reducing the dissociation rates of the complex (the *k*_*off*_ for the disassembly of the IscS/IscU complex is 0.8 s^−1^ in the absence of CyaY, vs. 0.006 s^−1^ in the presence of CyaY).

## A step forwards: molecular dynamic simulations

From our studies it emerged that the dynamic nature of the ISC proteins is a key factor in their functions. To feature this dynamical behavior, we thus complemented our previous data with extensive (400 ns) molecular dynamic (MD) simulations (di Maio et al., 2017) of the IscS-IscU complex, both in the presence and in the absence of CyaY. We showed that the binary IscS-IscU complex is stably folded in line with our SAXS evidence (Prischi et al., 2010a), but IscU adopts a likely functionally relevant pivotal motion around the interface with IscS. This means that, despite being firmly attached to IscS, IscU maintains some degree of flexibility upon complex formation, which can be connected to their low binding affinity and the need of IscU to deliver FeS cluster to protein acceptors. At the same time, the pivotal motions observed in the MD simulations suggest that IscS-IscU interface is “fluid,” with IscU side chains at the interface being trapped in several local minima. This was confirmed by NMR experiments (di Maio et al., 2017).

During the trajectory, the IscS catalytic loop containing Cys328 moves spontaneously and shifts from a mostly 3_10_-helical structure to a β-turn/3_10_-helix equilibrium, bringing Cys328 from 12 Å to 9 Å from the FeS cluster binding site on IscU (di Maio et al., 2017). This is in agreement with the IscS-IscU X-ray structure of the *A. fulgidus* (PDB ID 4EB5) (Marinoni et al., 2012), which brilliantly captured the delivery stage of FeS cluster from IscS to IscU. In 4EB5, the FeS cluster is bound to the Cys of the IscS catalytic loop and is about 6 Å away from the IscU FeS cluster binding site (Marinoni et al., 2012).

In agreement with our previous studies (Prischi et al., 2010a), the simulations showed that the tertiary complex IscS-IscU-CyaY is more stable and that CyaY reduces the structural fluctuations of the IscS-IscU complex (di Maio et al., 2017). The most striking feature of the complex is the absence of motions of the IscS catalytic loops (one for each protomer) over the same timescale, due to CyaY steric hindrance and a salt bridge between CyaY Arg53 and IscS Glu334. This model brings us back to the beginning of this review, where we described the possible roles of FXN. The model we generated recapitulated in an elegant way our enzymatic data and provides a mechanistic explanation of how CyaY slows down FeS cluster formation (Adinolfi et al., 2009; Prischi et al., 2010a).

## Extension of the methodology to other ISC complexes

**Figure 6:**
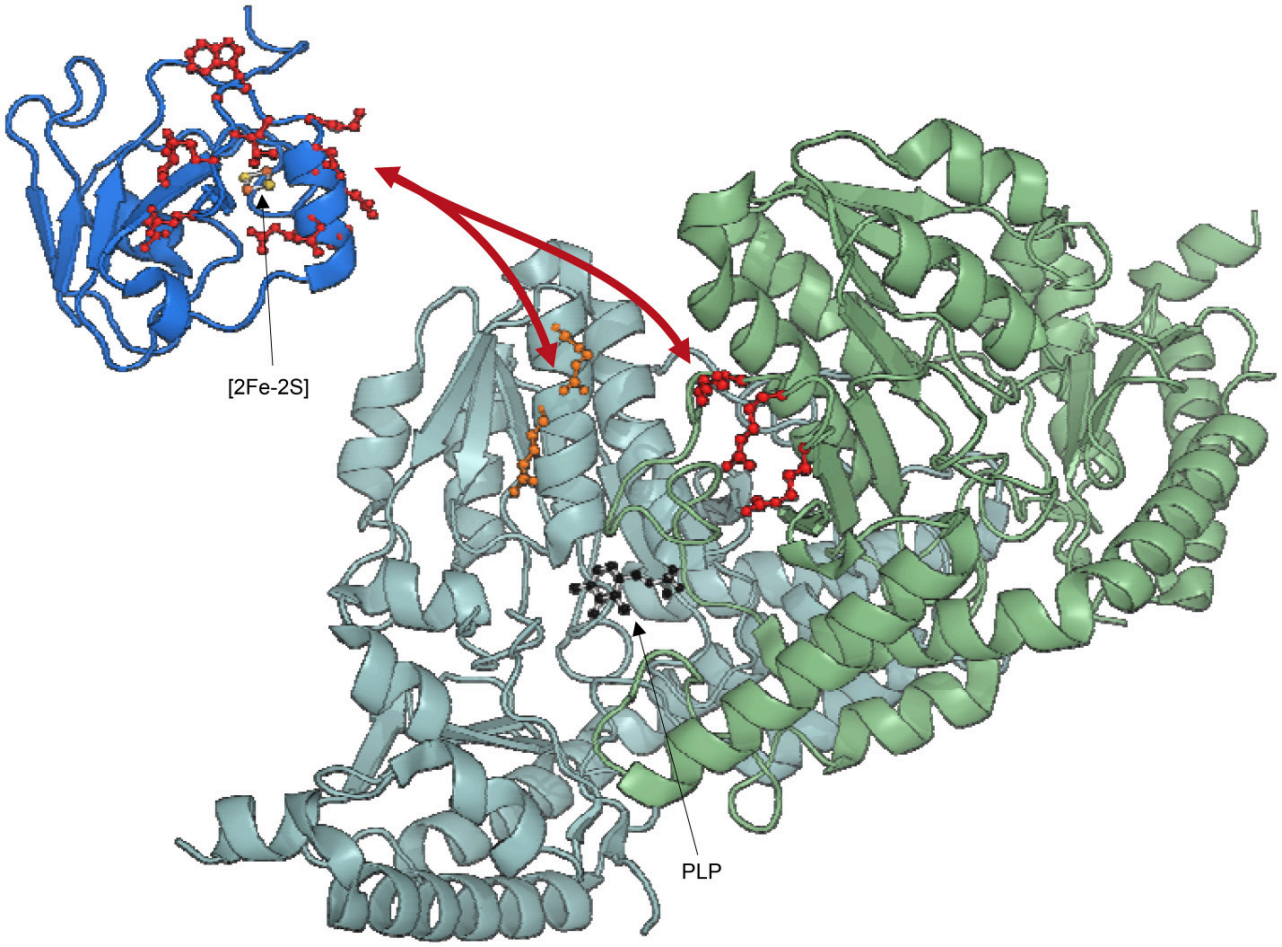
**Ribbon representation of Fdx, YfhJ, and IscS interaction**. IscS monomers are colored in pale cyan and light green with side chains of residues mutated indicated explicitly: R112E/R116E (orange), R220E/R223E/R225E (red). PLP in IscS active site is shown in black. Side chains of holo-Fdx residues exhibiting CSP (Ile54, Val55, Gln68, Glu69, Asp70, Asp71, Met72, Leu73, Asp74, Lys75, Ala76, Trp77, Gly78, Leu79, Glu80, Glu82) are explicitly shown in red. Fdx is loaded with a [2Fe-2S] cluster. YfhJ is colored in light blue and side chains of residues exhibiting CSP (Leu3, Lys4, Glu10, Ile11, Glu13, Ala14, Asp17, Leu58, Trp61, Leu62, Asp63, Glu64) are explicitly shown in blue.

The approach described here has now been adopted by others (Kim et al., 2014, 2015) also to elucidate other ISC complexes, increasing the robustness of the methodology. A study of the complex between IscS and YfhJ was published (Kim et al., 2014). YfhJ behaves similarly to CyaY as it is able to bind both Fe (II) and Fe (III) using an electrostatic negative surface, which is the same area involved in IscS binding (Figure 6; Pastore et al., 2006). YfhJ also competes for the same site of CyaY on IscS in agreement with previous mutation studies (Shi et al., 2010).

We have ourselves recently applied this hybrid method to model the IscS complex with Fdx, a FeS cluster dependent protein which is known to provide electrons to cellular reactions. Fdx is not-functional and devoid of tertiary structure in the absence of the cluster (Yan et al., 2013a). As for IscU, the spectrum of labeled Fdx disappears completely upon addition of unlabeled IscS. To circumvent the problem, we titrated ^2^H, ^15^N double-labeled holo-Fdx with IscS using [^2^H,^15^N]-SOFAST HMQC NMR experiments (Yan et al., 2013b). This experiment requires a shorter acquisition time compared to HSQC and is thus more suitable for unstable samples. We could then identify the residues of Fdx involved in IscS binding cluster, which reside near a uniform acidic patch on the α2-α3 loop (Figure 6; Yan et al., 2013b).

NMR competition studies revealed that Fdx and CyaY compete for the same site of IscS (Yan et al., 2013b). A Fdx-IscS SAXS verified model showed that Fdx sits in a position similar to that of CyaY near the active site. This was utterly validated by creating a new IscS mutant (IscS_R112E/R116E), which interferes with Fdx binding (Yan et al., 2013b). Assuming that the two proteins exploit their functions in different times during the cluster biogenesis, competition could represent a fascinating regulation mechanism. Superimposition of the Fdx-IscS and IscS-IscU models reveals that the Fdx C-terminus (which contains two key residues for electron transfer reactions, Tyr101 and His105) points toward the interface between IscS and IscU. This nicely explains how, after production of S^0^ from L-cysteine by IscS, Fdx could reduce S^0^ to S^2−^ (Yan et al., 2013b, 2015).

To add surprise to surprise, we have more recently shown that also the co-chaperone HscB binds to IscS in the same binding pocket, a result further validated by cross-linking experiments (Puglisi et al., 2016). This implies a picture in which IscS acts as a central platform on which several of the other bacterial ISC proteins bind and typically form 1:1 complexes (Pastore et al., 2006; Adinolfi et al., 2009; Prischi et al., 2010a; Yan et al., 2013b). It remains for us to understand why and how several different components of the same pathway compete for the same site. We suggested that this is a regulatory significance, which could operate through allosteric responses and involve the binding sites on each of the protomers present in the IscS dimer.

## Conclusions

In conclusions, we have in this review gone through a methodology (Table 1), which has allowed us to gain information on a 110 kDa complex with hybrid techniques. The method can in principle be applied also to larger complexes. The most successful cases are anyway those which involve an appreciable charge of shape of the complex, leading to a clear difference of the SAXS envelop between the isolated components and the complex. Limitations are currently dictated by the number of restraints available and by their distance tolerance: restraints which can allow a tolerance of more than 11 Å, as it is the case for cross-linking studies, are informative but only if several distances are available. It would also be useful to develop HADDOCK and other software to deal with the specific problems of hybrid methods. Some attempts along this line have already been made but more effort would be welcome in the future. It appears particularly useful, in a future perspective, to flank NMR and SAXS studies to other techniques, such as fluorescence, isothermal calorimetry, AUC and cross-linking to obtain more complete and complementary information. As a word of caution though, very good care should anyway be paid to the validation of the results. False positives can be easily obtained if assuming the presence of the wrong species in solution. It remains nevertheless clear that hybrid methods have open a new perspective to the size and complexity of the complexes which can be studied by Structural Biology and, more importantly to the possibility of tackle not only stable and rigid assemblies but also weakly interacting dynamical machines.

**Table 1:** **Hybrid method breakdown**.

	**PROS**	**CONS**
NMR	Proteins are in solution; Detects presence of protein interaction; Allows identification of residues involved in protein interaction; Solve structure of small complexes (<30 kDa) at atomic resolution; Allows to measure protein dynamics in solution	According to the exchange regime in the NMR time range it is not always possible to identify residues involved in interactions → Site Directed Mutagenesis can be used to modify affinity and hence the exchange regime; When the system crystallizes → X-Ray Crystallography and SAXS can be a valid alternative approach.
X-Ray Crystallography	Solves structure of a protein complex at atomic resolution.	Not all proteins or protein complexes crystallize.
SAXS	Proteins are in solution; Detects presence of protein interaction; Generate low resolution (10-20Å) models of proteins and protein complexes;	Requires 3D structures solved by NMR or X-Ray Crystallography; Unreliable protein complexes models built based only on SAXS data → Docking software (HADDOCK) can be used to generate models; Provides shape of the protein or protein complexes in solution; Generates reliable protein envelops only for monodisperse samples. Detect conformational changes.
Site directed mutagenesis	Allows to lower proteins affinities; Allows to abolish protein interactions.	May require the production of several different mutant clones in order to find residues involved in protein interaction; Does not provide overall structural information.
ITC & Fluorescence Spectroscopy	Detects presence of protein interaction; Measure affinities of protein complexes; → Allows to predict proteins exchange regime in the NMR time range.	Requires Site Directed Mutagenesis in order to identify residues involved in protein interactions; Does not provide overall structural information.
Protein-Protein Docking simulation (HADDOCK)	Generate 3D structures of protein complexes by forcing the protein interfaces to come together without imposing a particular orientation.	Requires 3D structures solved by NMR or X-Ray Crystallography; Reliable only in presence of experimental interaction restraints → NMR and Site Directed Mutagenesis can be used to identify residues involved in protein interaction.
Molecular Dynamics simulations	Allows to measure and observe dynamical features of proteins and proteins complexes.	Requires a 3D structure or an experimentally verified model.

*Flowchart of the pros and cons of the different techniques part of the hybrid method adopted for the study of Iron-sulfur cluster machinery*.

## Author contributions

All authors listed, have made substantial, direct and intellectual contribution to the work, and approved it for publication.

## Funding

The work was supported by MRC (U117584256).

### Conflict of interest statement

The authors declare that the research was conducted in the absence of any commercial or financial relationships that could be construed as a potential conflict of interest.
